# Air pollution exposure and pregnancy outcomes among women with polycystic ovary syndrome

**DOI:** 10.3389/fpubh.2022.1066899

**Published:** 2022-12-12

**Authors:** Qianqian Zhu, Jing Cai, Haiyan Guo, Yan Zhao, Jiaying Lin

**Affiliations:** ^1^Department of Assisted Reproduction, Shanghai Ninth People's Hospital, Jiao Tong University School of Medicine, Shanghai, China; ^2^Key Lab of Public Health Safety of the Ministry of Education, NHC Key Laboratory of Health Technology Assessment, School of Public Health, Fudan University, Shanghai, China; ^3^Shanghai Key Laboratory of Maternal Fetal Medicine, School of Medicine, Shanghai First Maternity and Infant Hospital, Tongji University, Shanghai, China

**Keywords:** air pollution, polycystic ovary syndrome, clinical pregnancy, live birth, reproductive age

## Abstract

**Background:**

Recently, the relationship between air pollution and reproductive outcomes has become a research focus. However, there is a lack of research on the relationship between air pollution and polycystic ovary syndrome (PCOS).

**Methods:**

This is a retrospective cohort study included a total of 1,652 women with PCOS and 12,543 women without PCOS conducted from 1 January 2015 to 31 December 2019. The average daily concentration data of six air pollutants (PM_2.5_, PM_10_, O_3_, NO_2_, SO_2_, and CO) during different exposure windows were obtained. Generalized estimating equation models were used to evaluate the association of air pollution with pregnancy outcomes.

**Results:**

Air pollutants were not found to have a significant association with pregnancy rates among patients with PCOS. However, each IQR increase in PM_10_ exposure during period 3 (embryo transfer to serum HCG test) was associated with the reduced clinical pregnancy rate (adjusted OR = 0.92, 95% CI: 0.84–0.99) for patients without PCOS. Patients without PCOS showed lower clinical pregnancy rates with increased exposure to NO_2_ during periods 2 (oocyte retrieval to embryo transfer) and 5 (start of gonadotropin medication to embryo transfer), with aORs and 95% CIs of 0.94 (0.88, 0.99) and 0.94 (0.88, 0.98), respectively. Each IQR increase in SO_2_ among patients without PCOS during periods 1 (start of gonadotropin medication to oocyte retrieval), 2, 5, and 6 (start of gonadotropin medication to serum HCG test) was related to a decrease in clinical pregnancy rate. For the live birth rate, no significant relationship was found between air pollutants, including PM_2.5_, PM_10_, SO_2_, NO_2_, CO, and O_3_, and the live birth rate for women with PCOS. However, women without PCOS presented a lower probability of live birth with exposure to SO_2_ during periods 1, 2, 5, and 6.

**Conclusion:**

This retrospective study of reproductive-aged women observed no significant relationships between ambient pollutants and pregnancy outcomes among women with PCOS but found negative associations among women without PCOS.

## Introduction

Polycystic ovary syndrome (PCOS) is a common endocrine disorder affecting about 5–20% of women of reproductive age (1). It is diagnosed by the presence of oligo- or anovulation, clinical and/or biochemical hyperandrogenism, and/or polycystic ovary morphology (2). PCOS is associated not only with reproductive manifestations (hyperandrogenism, anovulation, and infertility) but also with metabolic implications (dyslipidemia, type 2 diabetes, and potential cardiovascular disease) and psychological problems (anxiety, depression, and poor self-esteem), which causes heavy health and economic burden and is an important clinical and public health issue (1, 3–5). To date, the etiology of PCOS remains unclear. Both environmental and genetic factors are thought to contribute to the pathogenesis of PCOS.

Air pollution is one of the leading risk factors and a global environmental problem. Recently, the relationship between air pollution and reproductive outcomes has become a research focus (6–9). The exposure to air pollution has been reported to be related to the reduced fecundability and fertility rates or increased risk of infertility (10, 11). Ambient particulate matter in the air has been identified to adversely affect the sperm motility and induce the incidence of asthenozoospermia (12). The exposure to air pollution in pre-pregnancy and pregnancy stages has been found to associate with adverse pregnancy outcomes including lower clinical pregnancy rate and live birth rate and higher pregnancy loss rate (13, 14). However, there is a lack of research on the relationship between air pollution and PCOS. A cohort study conducted in Taiwan observed a high risk of PCOS in women with the increased exposure to fine air pollutants (15). A longitudinal cohort study of reproductive-aged women found no association of PM_2.5_ with polycystic ovary morphology, rather than PCOS (16). Whether ambient air pollution has more detrimental effects on pregnancy outcomes among women with PCOS than other women remains unknown.

With the rapid development of urbanization and industrialization, China has experienced serious air pollution, which has become the fourth largest threat to the Chinese people's health. In 2018, only 121 of 338 cities met the ambient air quality standards in China, and the main pollutant found in most of the key cities was PM_2.5_ (17). Considering the serious air pollution and common reproductive disorder of PCOS, large clinical studies investigating the association of air pollution with reproductive outcomes among women with PCOS are needed. Therefore, we carried out this research to compare the impact of air pollutant exposure on pregnancy outcomes of women with PCOS with that of women without PCOS in China.

## Materials and methods

### Study population

As PCOS is a common cause of female infertility, we conducted the present study among patients receiving assisted reproductive technology (ART) treatment. This retrospective cohort study was performed at the Assisted Reproduction Center of the Shanghai Ninth People's Hospital, affiliated with Jiao Tong University, School of Medicine (a large tertiary-care hospital in Shanghai, China). This study included a total of 1,652 women with PCOS and 12,543 women without PCOS receiving ART treatments from 1 January 2015 to 31 December 2019. The revised diagnostic criteria of the 2003 Rotterdam consensus were used to identify the disease of PCOS in our study, and patients were diagnosed with PCOS if they had at least two of the following three symptoms: oligo- or anovulation, clinical and/or biochemical signs of hyperandrogenism, and polycystic ovaries and exclusion of other etiologies, including congenital adrenal hyperplasia, androgen-secreting tumors, and Cushing's syndrome. The control group included patients diagnosed of tubal factor infertility or male factor infertility. The exclusion criteria included patients diagnosed with thyroid dysfunction, diabetes, hypertension, or tumors, as well as patients with congenital uterine malformations, unilateral oophorectomy, or chromosomal abnormalities. The Ethics Committee (Institutional Review Board) of the Shanghai Ninth People's Hospital has approved this study.

### IVF procedures

All the included patients underwent an *in vitro* fertilization (IVF) process involving four steps: controlled ovarian hyperstimulation (COH), oocyte retrieval, embryo transfer, and luteal support, as described in our previous published studies (18, 19). COH treatment was performed using the conventional stimulation protocol, the mild stimulation protocol, or progesterone-primed ovarian stimulation (PPOS) depending on the patients' age, body mass index, and ovarian reserve. When at least one follicle reached a diameter of ≥18 mm, human chorionic gonadotropin (HCG) was injected to trigger final oocyte maturation. Oocyte retrieval was performed 36 h after the HCG trigger. IVF or intracytoplasmic sperm injection (ICSI) was used for artificial fertilization according to semen parameters and clinical indications. Embryonic development was graded on days 2–3 according to the number and regularity of blastomeres and the percentage and pattern of anucleate fragments based on the Cummins criteria. For fresh embryo transfer, one or two fresh cleavage-stage or blastocyst-stage embryos were transferred on days 2–3 or days 5–6 after oocyte retrieval, whereas for frozen embryo transfer (FET), endometrial preparation was performed before embryo transfer with a natural cycle for regular menstrual cycles and a hormone therapy or stimulation cycle for irregular menstrual cycles. Then, one or two frozen-thawed embryos were transferred, and progesterone supplementation was provided until 8 weeks of gestation for patients with positive HCG tests.

The outcomes of this research included clinical pregnancy and live birth. Clinical pregnancy was defined as the observation of at least one gestational sac by vaginal ultrasound examination performed ~35 days after embryo transfer. A live birth was defined as at least one infant born alive after 24 weeks of gestation and survived more than 28 days.

### Air pollutant exposure assessment

Daily concentration data of six air pollutants, namely, inhalable particulate matter (PM_2.5_ and PM_10_), sulfur dioxide (SO_2_), nitrogen dioxide (NO_2_), carbon monoxide (CO), and ozone (O_3_), in the nearest monitoring station of each participant were obtained from the National Urban Air Quality Real-Time Publishing Platform, which is administered by the China National Environment Monitoring Center. The concentrations of the air pollutants for each patient were computed using the available data from the nearest monitoring station.

**Figure 1:**
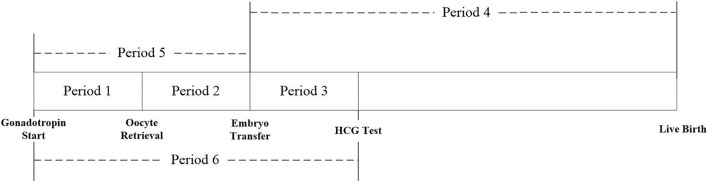
Timeline of IVF stage defined for this study.

This study evaluated the exposure to PM_2.5_, PM_10_, SO_2_, NO_2_, CO, and O_3_ for each patient during the different exposure periods of the IVF cycle: period 1: start of gonadotropin medication to oocyte retrieval; period 2: oocyte retrieval to embryo transfer; period 3: embryo transfer to serum HCG test; period 4: embryo transfer to delivery; period 5: start of gonadotropin medication to embryo transfer; and period 6: start of gonadotropin medication to serum HCG test (Figure 1).

### Statistical analysis

Patient characteristics were described by PCOS status (PCOS group/control group) using mean with standard deviation (SD) for continuous variables, and count with percentages for categorical variables. The concentrations of the ambient air pollutants during different exposure windows were summarized by mean, SD, median, and quartile range. The association of air pollution with IVF pregnancy outcomes (including clinical pregnancy and live birth rate) was examined using a logistic generalized estimating equation (GEE) model, considering the potential correlation between repeated embryo transfer cycles by the same patients as more than one cycle from the same patients were included. Air pollutants as exposure data were included in the model as categorical variables and were categorized into quartiles (Q_1_-Q_4_) based on their distribution and estimated ORs of pregnancy outcomes for Q_2_-Q_4_ compared with Q_1_. The covariates in the adjusted model included the patients' age, BMI, education level, infertility type, infertility duration, type of embryo transfer, number of embryos transferred, development stage of embryos transferred, fertilization method, endometrial thickness, and season of embryo transfer. Considering frozen embryo transfer was an important procedure of IVF, we also performed the multivariate analysis in a separate group. The patients' age, BMI, and number of embryo transfer cycles were important variables affecting the pregnancy outcomes of IVF, so we conducted sensitivity analyses in patients of patients younger than 35 years, those with normal BMI, and those receiving the first embryo transfer cycle. The results were compared with those of all participants to further explore the robustness of our findings. The unadjusted and adjusted odds ratios (ORs) and corresponding 95% confidence intervals (CIs) were reported for both PCOS and non-PCOS groups to assess the association of IVF pregnancy outcomes and neonatal outcomes with an interquartile range (IQR) increment of air pollutant exposure in different exposure periods. All statistical analyses were performed using the statistical package Stata, version 12 (StataCorp. Stata Statistical Software: Release 12. College Station, TX, USA) and a two-sided 5% level of significance.

## Results

### Participants' characteristics and ambient air pollutant exposure

A total of 1,652 women with PCOS undergoing 2,771 embryo transfer cycles and 12,543 women without PCOS undergoing 21,953 embryo transfer cycles were involved in the study. The detailed characteristics of the patients and their embryo transfer cycles in PCOS and non-PCOS groups are summarized in Table 1. The women with PCOS were younger, and their BMI was higher than the women without PCOS. More than 90% of embryo transfer cycles were frozen embryo transfers, the main fertilization method was IVF, and more than 80% were cleavage-stage embryo transfer for both patients with and without PCOS. Women with PCOS had a higher clinical pregnancy rate (55.86%) and live birth rate (44.93%) than women without PCOS (pregnancy rate: 45.93% and live birth rate: 37.53%).

**Table 1:** Demographic and clinical characteristics of 1,652 women with PCOS and 12,543 women without PCOS at baseline of ART cycles.

**Characteristics**	**PCOS group**	**Control group**	***P*** **-value**
**Personal characteristics**	1,652 women	12,543 women	
Female age (years)	29.49 ± 3.36	32.46 ± 5.10	< 0.001
<30	911 (55.15)	3941 (31.42)	
30–34	612 (37.05)	4,721 (37.64)	
≥35	129 (7.80)	3,881 (30.94)	
BMI (kg/m^2^)	23.95 ± 3.94	21.77 ± 2.98	< 0.001
Educational level			< 0.001
Less than senior high school	280 (16.95)	2,787 (22.22)	
Senior high school	251 (15.19)	2,037 (16.24)	
College or above	1,121 (67.86)	7,719 (61.54)	
Infertility type			< 0.001
Primary infertility	1,130 (68.40)	5,713 (45.55)	
Secondary infertility	522 (31.60)	6,830 (54.45)	
Duration of infertility (years)	3.17 ± 2.55	3.06 ± 2.97	0.188
**Cycle-specific characteristics**	2,771 cycles	21,953 cycles	
Type of embryo transfer			< 0.001
Fresh	102 (3.68)	1,447 (6.59)	
Frozen	2,669 (96.32)	20,506 (93.41)	
Fertilization method			< 0.001
IVF	1,559 (56.26)	14,680 (66.87)	
ICSI	583 (21.04)	5,842 (26.61)	
IVF + ICSI	629 (22.70)	1,431 (6.51)	
Number of embryos transferred			< 0.001
1	592 (21.36)	5,425 (24.71)	
2	2,179 (78.64)	16,528 (75.29)	
Development stage of embryos transferred			0.528
Cleavage stage	2,309 (83.33)	18,422 (83.92)	
Blastocyst	462 (16.67)	3,531 (16.08)	
Endometrial thickness	10.29 ± 2.32	10.57 ± 2.46	0.133
Season of embryo transfer			0.702
Spring	667 (24.07)	5,155 (23.48)	
Summer	831 (29.99)	6,591 (30.02)	
Autumn	788 (28.44)	6,457 (29.41)	
Winter	485 (17.50)	3,750 (17.08)	
Clinical pregnancy	1,548 (55.86)	10,082 (45.93)	< 0.001
Live birth	1,245 (44.93)	8,239 (37.53)	< 0.001

The average daily concentrations of the six ambient air pollutants in the different exposure periods for patients with and without PCOS are shown in Table 2. The average exposure concentrations of PM_2.5_, PM_10_, NO_2_, SO_2_, and CO in period 1 (start of gonadotropin medication to oocyte retrieval) were higher than those during other exposure windows for both women with and without PCOS. The daily concentrations of O_3_ in periods 2 (oocyte retrieval to embryo transfer) and 5 (start of gonadotropin medication to embryo transfer) for patients with or without PCOS were higher than those during other exposure windows.

**Table 2:** Distributions of six air pollutants during different exposure periods among women with and without PCOS.

	**PCOS**					**Control**				
**Pollutants**	**Mean**	**SD**	**25%**	**50%**	**75%**	**Mean**	**SD**	**25%**	**50%**	**75%**
PM_2.5_ (μg/m^3^)										
Period 1	44.43	21.38	28.45	40.84	54.58	46.35	22.28	30.89	42.53	55.97
Period 2	43.40	17.44	31.31	40.91	52.10	44.72	16.74	33.14	43.12	53.40
Period 3	41.78	21.56	27.08	36.87	51.28	43.00	21.64	27.98	38.13	52.67
Period 5	43.45	16.62	31.80	41.22	52.16	44.83	16.03	33.60	43.46	53.46
Period 6	43.43	16.27	32.16	41.18	52.07	44.83	15.71	33.85	43.47	53.27
PM_10_ (μg/m^3^)										
Period 1	71.72	32.34	48.21	65.77	87.44	73.68	32.68	51.44	67.53	87.69
Period 2	70.07	26.64	52.70	65.61	82.33	71.01	25.82	54.94	67.09	81.73
Period 3	68.23	32.81	45.27	61.37	81.85	69.00	31.89	46.90	62.29	82.48
Period 5	70.09	25.63	53.14	66.01	82.76	71.18	24.90	55.68	67.40	81.63
Period 6	70.04	25.28	53.38	65.61	81.95	71.15	24.45	55.80	67.44	81.27
O_3_ (μg/m^3^)										
Period 1	66.52	23.17	49.32	68.25	82.96	66.21	23.14	48.49	67.67	83.09
Period 2	67.70	17.84	55.47	68.24	79.43	66.93	17.57	55.44	67.50	77.58
Period 3	67.29	23.70	48.47	67.76	84.24	67.04	24.47	48.13	67.68	84.64
Period 5	67.71	16.84	56.00	68.36	79.23	66.92	16.61	55.79	67.65	77.37
Period 6	67.47	16.28	56.20	68.00	78.48	66.76	16.08	56.02	67.43	76.78
NO_2_ (μg/m^3^)										
Period 1	35.49	14.73	23.72	33.72	45.20	36.32	14.88	25.09	34.72	46.35
Period 2	34.38	11.62	26.15	34.49	42.65	35.02	11.55	26.31	35.70	43.60
Period 3	34.15	14.25	23.43	32.36	43.73	34.85	14.23	24.09	33.19	44.53
Period 5	34.43	11.35	26.12	34.74	42.94	35.11	11.23	26.63	35.99	43.60
Period 6	34.46	11.16	26.44	34.96	42.64	35.13	11.04	26.79	36.06	43.55
SO_2_ (μg/m^3^)										
Period 1	15.04	8.33	9.45	13.08	18.38	16.37	10.06	10.02	13.90	19.75
Period 2	14.13	6.96	9.23	12.81	17.43	15.22	7.92	10.11	13.78	18.46
Period 3	13.32	7.48	8.36	11.67	16.41	14.14	8.44	8.77	12.26	17.15
Period 5	14.19	6.88	9.37	12.93	17.53	15.30	7.81	10.28	13.87	18.50
Period 6	14.15	6.79	9.38	12.90	17.51	15.26	7.73	10.25	13.86	18.43
CO (mg/m^3^)										
Period 1	0.84	0.26	0.66	0.78	0.97	0.85	0.28	0.68	0.80	0.98
Period 2	0.82	0.22	0.69	0.78	0.91	0.84	0.22	0.71	0.81	0.92
Period 3	0.81	0.28	0.64	0.75	0.91	0.82	0.27	0.65	0.76	0.94
Period 5	0.82	0.22	0.69	0.78	0.91	0.84	0.22	0.71	0.81	0.92
Period 6	0.82	0.21	0.69	0.78	0.91	0.84	0.21	0.71	0.81	0.92

SD, standard deviation; P_25_, P_50_, P_75_: 25th, 50th, and 75th percentile.

PM_2.5_, particulate matter ≤ 2.5 μm, μg/m^3^; PM_10_, particulate matter ≤ 10 μm, μg/m^3^; NO_2_, nitrogen dioxide, μg/m^3^; SO_2_, sulfur dioxide, μg/m^3^; CO, carbon monoxide, mg/m^3^; O_3_, ozone, μg/m^3^.

Periods 1, 2, 3, 5, and 6 indicate the exposure windows shown in Figure 1.

Regarding the interrelationship of air pollutants during the IVF treatment for both PCOS and control groups, most air pollutants were positively related to each other, except O_3_, which was negatively correlated with other pollutants (Supplementary Table S3).

### Exposure to ambient air pollutants and IVF pregnancy outcomes

Table 3 presents the relationships between air pollutants with pregnancy outcomes for women with and without PCOS. For clinical pregnancy, air pollutant was not significantly associated with the pregnancy rate among patients with PCOS. However, the result showed each IQR increase of PM_10_ exposure in period 3 (embryo transfer to serum HCG test) was significantly associated with an 8% lower clinical pregnancy rate (adjusted OR = 0.92, 95% CI: 0.84–0.99) for patients without PCOS. Patients without PCOS showed a lower clinical pregnancy rate with increased exposure to NO_2_ during periods 2 (oocyte retrieval to embryo transfer) and 5 (start of gonadotropin medication to embryo transfer) with aORs and 95% CIs of 0.94 (0.88, 0.99) and 0.94 (0.88, 0.98), respectively. Each IQR increase in SO_2_ among patients without PCOS during periods 1 (start of gonadotropin medication to oocyte retrieval), 2 (oocyte retrieval to embryo transfer), 5 (start of gonadotropin medication to embryo transfer), and 6 (start of gonadotropin medication to serum HCG test) was significantly related to a decrease in the clinical pregnancy rate, with aORs and 95% CIs of 0.94 (0.90,0.97), 0.94 (0.90,0.98), 0.94 (0.90,0.97), and 0.94 (0.90,0.98), respectively.

**Table 3:** Association between ambient air pollution exposure and pregnancy outcome and live birth among women with and without PCOS.

		**Clinical pregnancy**				**Live birth**			
**Pollutants**	**Exposure period**	**PCOS**		**Control**		**PCOS**		**Control**	
		**aOR (95% CI)**	***P*** **-value**	**aOR (95% CI)**	***P*** **-value**	**aOR (95% CI)**	***P*** **-value**	**aOR (95% CI)**	***P*** **-value**
PM_2.5_	Period 1	1.10 (0.84, 1.43)	0.502	1.02 (0.93, 1.11)	0.717	1.07 (0.84, 1.37)	0.568	1.02 (0.94, 1.12)	0.620
	Period 2	1.24 (0.92, 1.67)	0.151	1.05 (0.95, 1.16)	0.292	1.06 (0.80, 1.40)	0.683	1.04 (0.94, 1.15)	0.399
	Period 3	1.17 (0.86, 1.60)	0.310	1.04 (0.94, 1.14)	0.443	1.12 (0.84, 1.49)	0.447	1.02 (0.93, 1.13)	0.683
	Period 5	1.26 (0.93, 1.72)	0.138	1.05 (0.95, 1.16)	0.368	1.12 (0.83, 1.50)	0.462	1.02 (0.92, 1.14)	0.655
	Period 6	1.31 (0.95, 1.82)	0.101	1.04 (0.94, 1.16)	0.407	1.12 (0.83, 1.53)	0.456	1.03 (0.92, 1.14)	0.640
PM_10_	Period 1	0.99 (0.78, 1.25)	0.922	1.00 (0.91, 1.08)	0.921	0.98 (0.79, 1.22)	0.840	0.99 (0.91, 1.07)	0.766
	Period 2	0.84 (0.65, 1.08)	0.169	0.97 (0.90, 1.05)	0.471	1.01 (0.79, 1.29)	0.946	0.98 (0.90, 1.06)	0.568
	Period 3	0.76 (0.57, 1.02)	0.069	**0.92 (0.84, 0.99)**	**0.040**	0.84 (0.64, 1.11)	0.222	0.96 (0.89, 1.05)	0.395
	Period 5	0.84 (0.64, 1.11)	0.218	0.97 (0.90, 1.06)	0.547	0.96 (0.74, 1.25)	0.770	0.98 (0.91, 1.07)	0.746
	Period 6	0.81 (0.60, 1.07)	0.142	0.97 (0.90, 1.05)	0.487	0.95 (0.72, 1.24)	0.707	0.98 (0.91, 1.07)	0.697
O_3_	Period 1	1.06 (0.91, 1.23)	0.457	0.96 (0.91, 1.01)	0.124	1.14 (0.99, 1.32)	0.068	0.97 (0.92, 1.02)	0.237
	Period 2	0.95 (0.82, 1.10)	0.476	1.01 (0.97, 1.06)	0.548	0.94 (0.81, 1.08)	0.375	1.03 (0.98, 1.08)	0.213
	Period 3	1.11 (0.93, 1.32)	0.245	1.00 (0.95, 1.06)	0.990	1.12 (0.95, 1.31)	0.161	1.00 (0.94, 1.06)	0.909
	Period 5	0.99 (0.85, 1.16)	0.926	0.99 (0.95, 1.05)	0.953	1.01 (0.86, 1.17)	0.928	1.02 (0.97, 1.07)	0.519
	Period 6	1.01 (0.87, 1.18)	0.867	1.00 (0.95, 1.05)	0.980	1.02 (0.88, 1.18)	0.813	1.02 (0.97, 1.06)	0.530
NO_2_	Period 1	1.06 (0.88, 1.26)	0.553	0.99 (0.94, 1.06)	0.895	1.06 (0.89, 1.26)	0.499	0.99 (0.93, 1.06)	0.808
	Period 2	0.98 (0.83, 1.16)	0.811	**0.94 (0.88, 0.99)**	**0.048**	0.99 (0.84, 1.17)	0.928	0.95 (0.89, 1.02)	0.140
	Period 3	0.98 (0.82, 1.17)	0.815	0.97 (0.91, 1.04)	0.396	0.95 (0.80, 1.13)	0.538	0.98 (0.92, 1.04)	0.507
	Period 5	0.97 (0.81, 1.16)	0.756	**0.94 (0.88, 0.98)**	**0.046**	0.99 (0.83, 1.17)	0.901	0.95 (0.89, 1.02)	0.162
	Period 6	0.96 (0.81, 1.14)	0.629	0.94 (0.88, 1.00)	0.061	0.98 (0.83, 1.16)	0.792	0.96 (0.90, 1.02)	0.166
SO_2_	Period 1	0.94 (0.84, 1.06)	0.310	**0.94 (0.90, 0.97)**	**0.001**	1.01 (0.90, 1.13)	0.846	**0.93 (0.90, 0.97)**	**0.001**
	Period 2	0.97 (0.85, 1.12)	0.742	**0.94 (0.90, 0.98)**	**0.003**	0.96 (0.83, 1.10)	0.522	**0.94 (0.90, 0.98)**	**0.009**
	Period 3	1.04 (0.90, 1.20)	0.585	0.97 (0.94, 1.01)	0.217	1.00 (0.88, 1.14)	0.950	0.97 (0.93, 1.01)	0.095
	Period 5	0.97 (0.84, 1.12)	0.673	**0.94 (0.90, 0.97)**	**0.003**	0.97 (0.84, 1.11)	0.642	**0.94 (0.90, 0.98)**	**0.008**
	Period 6	0.98 (0.85, 1.14)	0.821	**0.94 (0.90, 0.98)**	**0.005**	0.98 (0.85, 1.13)	0.752	**0.94 (0.90, 0.99)**	**0.010**
CO	Period 1	1.00 (0.87, 1.16)	0.952	0.99 (0.95, 1.04)	0.737	0.98 (0.85, 1.12)	0.731	0.99 (0.95, 1.04)	0.854
	Period 2	0.97 (0.85, 1.11)	0.671	1.01 (0.96, 1.05)	0.745	0.97 (0.85, 1.10)	0.626	1.00 (0.96, 1.04)	0.988
	Period 3	0.98 (0.86, 1.13)	0.808	1.03 (0.98, 1.08)	0.228	1.01 (0.89, 1.15)	0.838	1.01 (0.96, 1.06)	0.742
	Period 5	0.98 (0.85, 1.12)	0.736	1.00 (0.96, 1.04)	0.960	0.98 (0.86, 1.12)	0.768	0.99 (0.95, 1.04)	0.867
	Period 6	0.98 (0.86, 1.12)	0.747	1.00 (0.96, 1.05)	0.865	0.98 (0.87, 1.11)	0.788	0.99 (0.95, 1.04)	0.921

Results shown in bold indicate statistically significant associations.

PM_2.5_, particulate matter ≤ 2.5μm, μg/m^3^; PM_10_, particulate matter ≤ 10μm, μg/m^3^; NO_2_, nitrogen dioxide, μg/m^3^; SO_2_, sulfur dioxide, μg/m^3^; CO, carbon monoxide, mg/m^3^; O_3_, ozone, μg/m^3^.

Model adjusted for patients' age (<30, 30-34, 35-39, and ≥40 years), BMI, education level, infertility type (primary or secondary infertility), infertility duration, infertility cause (female factor, male factor, both, and unexplained factor), type of embryo transfer (fresh or frozen embryo transfer), number of embryos transferred (one or two), fertilization method (IVF, ICSI, or IVF + ICSI), endometrial thickness, and season of embryo transfer (spring, summer, autumn, or winter).

Periods 1, 2, 3, 5, and 6 indicate the exposure windows shown in Figure 1.

For the live birth rate, no significant relationship was found between the ambient air pollutants, namely, PM_2.5_, PM_10_, SO_2_, NO_2_, CO, and O_3_, and the live birth rate for women with PCOS (Table 3). However, we observed women without PCOS had a lower live birth rate with higher exposure to SO_2_ during periods 1 (start of gonadotropin medication to oocyte retrieval), 2 (oocyte retrieval to embryo transfer), 5 (start of gonadotropin medication to embryo transfer), and 6 (start of gonadotropin medication to serum HCG test), with aORs and 95% CIs of 0.93 (0.90,0.97), 0.94 (0.90,0.98), 0.94 (0.90,0.98), and 0.94 (0.90,0.99), respectively.

**Figure 2:**
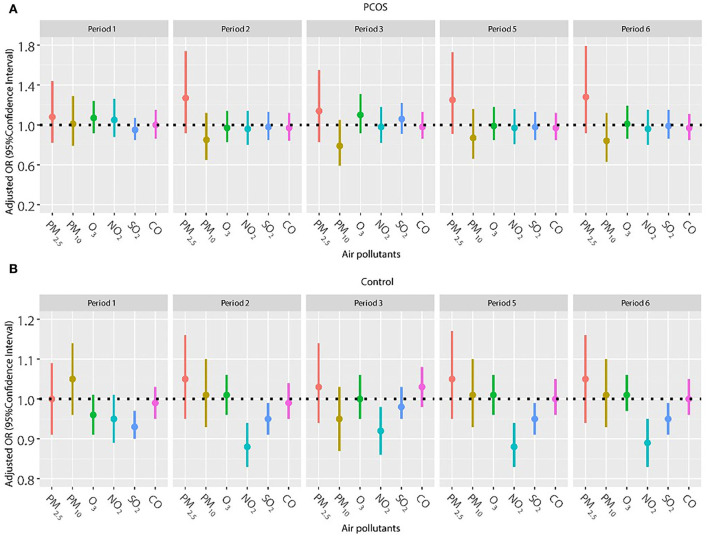
**(A,B)** Association between ambient air pollution exposure and clinical pregnancy rate among women with and without PCOS with frozen embryo transfer. Results showed in bold indicated statistically significant associations. PM_2.5_, particulate matter ≤ 2.5μm, μg/m^3^; PM_10_, particulate matter ≤ 10 μm, μg/m^3^; NO_2_, nitrogen dioxide, μg/m^3^; SO_2_, sulfur dioxide, μg/m^3^; CO, carbon monoxide, mg/m^3^; O_3_, ozone, μg/m^3^. Model adjusted for patients' age (<30, 30–34, 35–39, and ≥40 years), BMI, education level, infertility type (primary or secondary infertility), infertility duration, infertility cause (female factor, male factor, both, and unexplained factor), number of embryos transferred (one or two), fertilization method (IVF, ICSI, or IVF + ICSI), endometrial thickness, and season of embryo transfer (spring, summer, autumn, or winter). Periods 1, 2, 3, 5, and 6 indicate the exposure windows shown in Figure 1.

**Figure 3:**
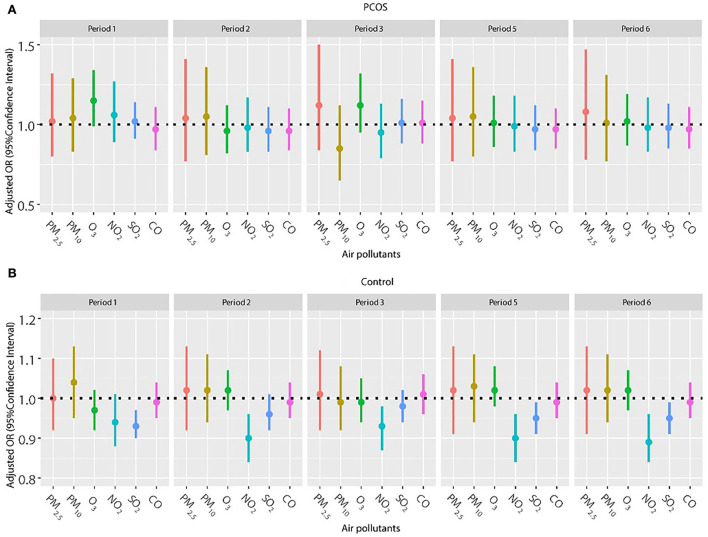
**(A,B)** Association between ambient air pollution exposure and live birth rate among women with and without PCOS with frozen embryo transfer. Results showed in bold indicated statistically significant associations. PM_2.5_, particulate matter ≤ 2.5 μm, μg/m^3^; PM_10_, particulate matter ≤ 10 μm, μg/m^3^; NO_2_, nitrogen dioxide, μg/m^3^; SO_2_, sulfur dioxide, μg/m^3^; CO, carbon monoxide, mg/m^3^; O_3_, ozone, μg/m^3^. Model adjusted for patients' age (<30, 30–34, 35–39, and ≥40 years), BMI, education level, infertility type (primary or secondary infertility), infertility duration, infertility cause (female factor, male factor, both, and unexplained factor), number of embryos transferred (one or two), fertilization method (IVF, ICSI, or IVF + ICSI), endometrial thickness, and season of embryo transfer (spring, summer, autumn, or winter). Periods 1, 2, 3, 5, and 6 indicate the exposure windows shown in Figure 1.

Frozen embryo transfer has been widely used in women with PCOS to improve the pregnancy outcome, so we also explored the association of air pollutants and pregnancy outcomes among women with frozen embryo transfer (Figures 2, 3). The results did not show a significant relationship between any air pollutants and clinical pregnancy or live birth in women with PCOS in the frozen embryo transfer cycles. Conversely, increased exposure to NO_2_ was related to decreased probability of clinical pregnancy and live birth among women without PCOS during periods 2 (oocyte retrieval to embryo transfer), 3 (embryo transfer to serum HCG test), 5 (start of gonadotropin medication to embryo transfer), and 6 (start of gonadotropin medication to serum HCG test). In addition, higher exposure to SO_2_ was associated with a reduced probability of clinical pregnancy for women without PCOS during periods 1 (start of gonadotropin medication to oocyte retrieval), 5 (start of gonadotropin medication to embryo transfer), and 6 (start of gonadotropin medication to serum HCG test).

**Figure 4:**
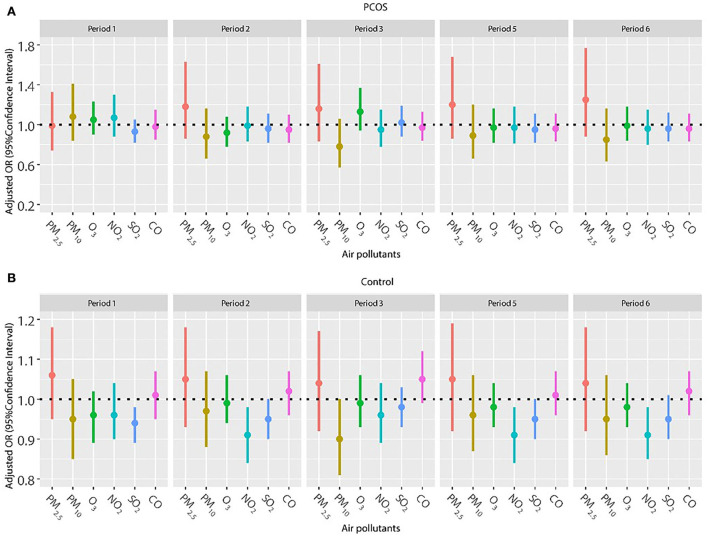
**(A,B)** Association between ambient air pollution exposure and clinical pregnancy rate among women with and without PCOS younger than 35 years. Results showed in bold indicated statistically significant associations. PM_2.5_, particulate matter ≤ 2.5 μm, μg/m^3^; PM_10_, particulate matter ≤ 10 μm, μg/m^3^; NO_2_, nitrogen dioxide, μg/m^3^; SO_2_, sulfur dioxide, μg/m^3^; CO, carbon monoxide, mg/m^3^; O_3_, ozone, μg/m^3^. Model adjusted for BMI, education level, infertility type (primary or secondary infertility), infertility duration, infertility cause (female factor, male factor, both, and unexplained factor), type of embryo transfer (fresh or frozen embryo transfer), number of embryos transferred (one or two), fertilization method (IVF, ICSI, or IVF + ICSI), endometrial thickness, and season of embryo transfer (spring, summer, autumn, or winter). Periods 1, 2, 3, 5, and 6 indicate the exposure windows shown in Figure 1.

**Figure 5:**
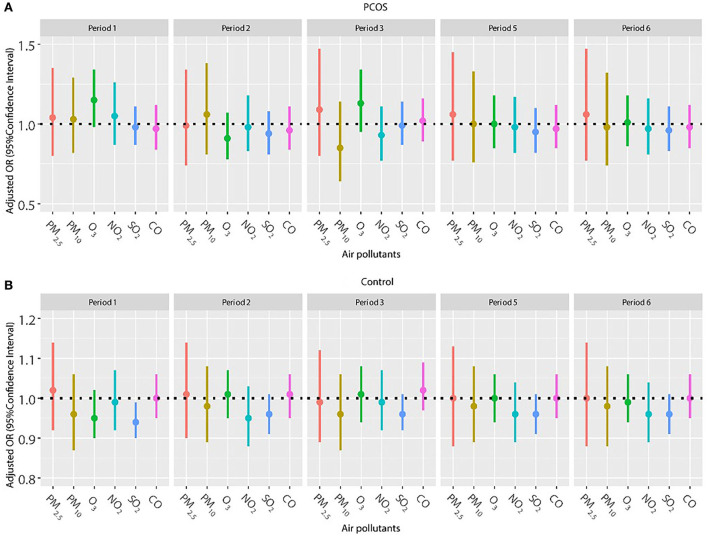
**(A,B)** Association between ambient air pollution exposure and live birth rate among PCOS and non-PCOS women aged less than 35 years. Note: Results showed in bold indicated statistically significant associations. PM^2.5^, particulate matter ≤ 2.5μm, μg/m^3^; PM10, particulate matter ≤ 10μm, μg/m^3^; NO_2_, nitrogen dioxide, μg/m^3^; SO_2_, sulfur dioxide, μg/m^3^; CO, carbon monoxide, mg/m^3^; O_3_, ozone, μg/m3. Model adjusted for BMI, education level, infertility type (primary or secondary infertility), infertility duration, infertility cause (female factor, male factor, both, and unexplained factor), type of embryo transfer (fresh or frozen embryo transfer), number of embryos transferred (one or two), fertilization method (IVF, ICSI or IVF+ICSI), endometrial thickness, and season of embryo transfer (spring, summer, autumn, or winter). Period 1, 2, 3, 5, 6 indicate the exposure windows shown in Figure 1.

Considering a higher proportion of older women in the control group and the important effect of age on pregnancy outcomes, we repeated the analysis in patients younger than 35 years (Figures 4, 5). The results were in line with those of the whole population. No significant relationship was found between air pollutants, and clinical pregnancy and live birth among women with PCOS. However, adverse effects of NO_2_ on clinical pregnancy during periods 2 (oocyte retrieval to embryo transfer), 5 (start of gonadotropin medication to embryo transfer), and 6 (start of gonadotropin medication to serum HCG test) were found among women without PCOS. In addition, ambient SO_2_ was also correlated with a lower pregnancy rate or live birth rate in period 1 (start of gonadotropin medication to oocyte retrieval) among women without PCOS.

The sensitivity analyses were restricted to women with normal BMI and women with the first embryo transfer cycle, and no significant change was found. Among women with normal BMI or women with the first embryo transfer cycle, air pollutants were not found to be significantly related to pregnancy or live birth rates for women with PCOS. However, negative relationships between NO_2_ and SO_2_, and pregnancy or live birth rate were found for women without PCOS with normal BMI or with the first embryo transfer cycle (Supplementary Tables S1, S2).

## Discussion

### Principal findings

To our knowledge, this is the first epidemiological study exploring the relationship between air pollutants and pregnancy outcomes among women with PCOS. We found no significant relationships between ambient pollutants and pregnancy outcomes among women with PCOS but negative associations of air pollutants with clinical pregnancy or live birth for women without PCOS. The aforementioned different relationships between ambient pollutants and pregnancy outcomes of patients with and without PCOS existed even after analyses were restricted to patients younger than 35 years, patients undergoing frozen embryo transfer, patients with the first embryo transfer cycles, or patients with normal weight accounting for potential confounders.

### Clinical and research implications

The PCOS is a common complex condition associated with endocrine, metabolic, psychological, and reproductive features (20). As a global epidemic of chronic disease, PCOS causes a heavy health and socioeconomic burden globally. The etiology of PCOS involves genetic, environmental, and lifestyle factors (21). Strong and compelling scientific evidence demonstrated the important role of environmental factors in the pathogenesis of PCOS and a <10% contribution for genetic factors (22). There is a wealth of evidence from human epidemiological research suggesting environment factors are of clinical importance for the pathogenesis of PCOS. However, almost all studies focused on endocrine-disrupting chemicals (EDCs), with only few on air pollutants. A previous study has reported the elevated levels of BPA in patients with PCOS in comparison to healthy controls (23). The study performed by Yang et al. also measured other EDCs including perfluorooctanoate, perfluorooctane sulfonate, polychlorinated biphenyls, and polycyclic aromatic hydrocarbons in patients with PCOS and control patients. The result showed a higher serum concentration of the aforementioned pollutants in the PCOS group than in the control group (24). Studies have suggested EDCs may disrupt several neuroendocrine, hormonal, and metabolic signaling pathways, leading to the clinical features of PCOS (25, 26). Considering the widespread nature of EDCs in the environment and consumer products, it has been advised to reduce the use of EDCs to protect women with PCOS from increasing adverse health effects. Air pollution has also been one of the most concerned environmental health problems. However, the current research on the correlation of PCOS with air pollution was limited. Air pollution has also been assumed to interfere with the female reproductive system due to the disturbance of hormone homeostasis. Only one prospective study by Lin et al. conducted in Taiwanese observed that the fourth quartile levels of exposure to air pollutants including SO_2_, NO, NO_2_, NO_x_, and PM_2.5_ increased the risk of PCOS compared with the first-quartile levels of exposure (15). The authors speculated that air pollutants may lead to active oxidative stress, DNA damage, mitochondrial damage, and thus predispose to develop PCOS. In this epidemiological study, we demonstrated a higher exposure to air pollutants increased the risk of PCOS but did not explore its effects on reproductive outcomes including pregnancy or live birth of women with PCOS. For women with PCOS, the main concern was whether they will have a successful conception and delivery. So, exploring the influence of air pollutants on reproductive outcomes of women with PCOS has important clinical and scientific implications.

Since air pollution is considered a global health threat, the association of air pollutants with reproductive outcomes has become a research focus (6, 7, 9). In our study, we observed a lower pregnancy rate with increased exposure to PM_10_, NO_2_, or SO_2_, and decreased probability of live birth with exposure to SO_2_ among patients without PCOS. These results are consistent with the results of some previous studies. A retrospective study reported the exposure to PM_2.5_ or PM_10_ before the period of oocyte retrieval adversely affected pregnancy and live birth rates in the general population undergoing IVF (27). Another study from Shanghai by Shi found a higher exposure to PM_10_, NO_2_, and SO_2_ was related to reduced pregnancy or live birth rates in general patients with IVF (28). However, contrary to these results, significant correlations of air pollutants with clinical pregnancy and live birth rates were not observed in patients with PCOS in this study. Considering that research on this topic is scarce, further research is needed to support these findings. We speculate that significant differences in susceptibility to air pollutants may explain the inconsistent results between patients with and without PCOS for the influences of air pollutions on reproductive outcomes. A previous research study has provided scientific evidence that air pollutants play an important role in the incidence of PCOS (22). PCOS patients are not vulnerable to the impact of air pollutants on reproductive outcomes compared with the general patients. The non-PCOS patients were more susceptible to air pollutions than PCOS patients for the health influences. We speculate air pollutants may lead to changes in susceptible gene expression related to the pathogenesis of PCOS. The underlying mechanism needs to be studied further.

### Strengths and limitations

This study has several strengths. The present study included a large number of patients with PCOS, which, to our knowledge, was the first large study performed to explore associations of air pollutants with reproductive outcomes among patients with PCOS. We expect this study to fill the gap in the literature by estimating the potential impact of air pollutant exposure on reproductive outcomes in different clinical states, such as patients with PCOS. With the rapid development of cryopreservation techniques, frozen embryo transfer has become an alternative to fresh embryo transfer in ART treatment, especially for patients with PCOS, and it may reduce the risk of ovarian hyperstimulation syndrome (19). A large amount of data related to frozen embryo transfer allowed us to examine the aforementioned relationship in frozen embryo transfer cycles, which may enrich currently existing research. Results from previous studies revealed the relationship between air pollutions and health outcomes was stronger among women with certain characteristics (e.g., women living in rural areas and women younger than 32 years), which indicated women with certain characteristics tend to be more sensitive to air pollutions (28). More convincing results of the relationship between air pollution and reproductive outcomes for women with PCOS were obtained from this research when the analysis was performed in women younger than 35 years, normal BMI women, or women with first embryo transfer cycles.

The present study also has some limitations. First, this was a retrospective study; however, this study had high-quality data by using strict inclusion criteria. Second, air pollution exposure data from nearest monitoring stations of participants were used, instead of personal exposure, which may lead to the non-differential exposure misclassification (29). Third, although a variety of potential confounders were adjusted in our study, other confounding factors such as nutrition conditions, socioeconomic conditions, and some health-seeking practices, as well as adherence to the IVF protocol, were not considered. Finally, this study was performed among women receiving IVF treatment, which restricts the generalizability of the findings to all women of reproductive age. Based on these limitations, large prospective cohort studies among women of reproductive age from the general population should be carried out in future to confirm these findings.

## Conclusion

This retrospective cohort study including a large number of women with PCOS did not observe significant relationships between ambient pollutants and pregnancy outcomes. However, negative associations of ambient air pollutants with clinical pregnancy and live birth were found for women without PCOS. Moreover, the aforementioned different relationships between ambient pollutants and pregnancy outcomes in patients with and without PCOS existed when analyses were restricted to patients younger than 35 years, patients with frozen embryo transfer, patients with the first embryo transfer cycles, or patients with normal weight. Large prospective cohort studies among reproductive-aged women from the general population are needed to explore the underlying mechanism.

## Data availability statement

The original contributions presented in the study are included in the article/Supplementary material, further inquiries can be directed to the corresponding author.

## Ethics statement

The studies involving human participants were reviewed and approved by the Ethics Committee (Institutional Review Board) of the Shanghai Ninth People's Hospital. The patients/participants provided their written informed consent to participate in this study.

## Author contributions

QZ, JC, and JL designed the study and drafted the manuscript. YZ, HG, and JC conducted data acquisition and statistical analysis. QZ and JL revised the manuscript. All authors approved the final manuscript.
