# Supplementary material for: Air pollution exposure and pregnancy outcomes among women with polycystic ovary syndrome
Source: Front Public Health. 2022 Dec 12;10:1066899. doi: 10.3389/fpubh.2022.1066899 (PMC9791261; doi:10.3389/fpubh.2022.1066899)
Supplement: Supplementary file 1 [file Data_Sheet_1.docx]

Table S1 Association between ambient air pollution exposure and pregnancy outcome and live birth among PCOS and non-PCOS women with normal BMI

|  |  | Clinical pregnancy | | | | Live birth | | | |
| --- | --- | --- | --- | --- | --- | --- | --- | --- | --- |
| Pollutants | Exposure period | PCOS | | Control | | PCOS | | Control | |
|  |  | aOR(95%CI) | P-value | aOR(95%CI) | P-value | aOR(95%CI) | P-value | aOR(95%CI) | P-value |
| PM_2.5_ | Period 1 | 0.84(0.60,1.18) | 0.316 | 1.04(0.94,1.15) | 0.457 | 0.87(0.64,1.17) | 0.354 | 1.03(0.93,1.13) | 0.605 |
|  | Period 2 | 1.21(0.83,1.76) | 0.320 | 1.08(0.97,1.20) | 0.178 | 1.02(0.84,1.23) | 0.871 | 1.08(0.96,1.20) | 0.197 |
|  | Period 3 | 1.03(0.69,1.53) | 0.887 | 1.04(0.93,1.15) | 0.487 | 1.02(0.72,1.45) | 0.895 | 1.05(0.95,1.17) | 0.351 |
|  | Period 5 | 1.20(0.81,1.79) | 0.364 | 1.09(0.98,1.23) | 0.119 | 0.92(0.64,1.34) | 0.667 | 1.07(0.95,1.20) | 0.272 |
|  | Period 6 | 1.28(0.84,1.94) | 0.254 | 1.09(0.97,1.23) | 0.137 | 0.95(0.65,1.39) | 0.792 | 1.07(0.95,1.20) | 0.259 |
| PM_10_ | Period 1 | 1.29(0.96,1.74) | 0.085 | 1.03(0.93,1.13) | 0.611 | 1.28(0.98,1.67) | 0.071 | 1.03(0.94,1.14) | 0.486 |
|  | Period 2 | 0.88(0.64,1.19) | 0.404 | 1.01(0.92,1.10) | 0.884 | 1.07(0.89,1.29) | 0.474 | 1.00(0.92,1.10) | 0.932 |
|  | Period 3 | 0.88(0.62,1.25) | 0.484 | 0.94(0.86,1.03) | 0.214 | 0.96(0.70,1.33) | 0.823 | 0.97(0.89,1.06) | 0.538 |
|  | Period 5 | 0.92(0.66,1.28) | 0.626 | 0.99(0.91,1.09) | 0.979 | 1.15(0.85,1.56) | 0.371 | 1.09(0.92,1.11) | 0.850 |
|  | Period 6 | 0.86(0.61,1.21) | 0.389 | 0.99(0.90,1.09) | 0.848 | 1.12(0.82,1.52) | 0.485 | 1.00(0.91,1.10) | 0.967 |
| O_3_ | Period 1 | 1.02(0.84,1.23) | 0.877 | 0.97(0.91,1.02) | 0.248 | 1.13(0.94,1.35) | 0.195 | 0.98(0.93,1.04) | 0.618 |
|  | Period 2 | 0.96(0.78,1.19) | 0.713 | 1.00(0.95,1.05) | 0.959 | 0.92(0.76,1.11) | 0.381 | 1.03(0.98,1.09) | 0.240 |
|  | Period 3 | 1.15(0.92,1.44) | 0.216 | 1.02(0.96,1.09) | 0.562 | 1.12(0.91,1.37) | 0.284 | 1.01(0.95,1.08) | 0.634 |
|  | Period 5 | 0.96(0.77,1.20) | 0.715 | 0.99(0.95,1.05) | 0.901 | 0.96(0.79,1.18) | 0.732 | 1.03(0.97,1.08) | 0.354 |
|  | Period 6 | 1.00(0.80,1.24) | 0.973 | 1.00(0.95,1.06) | 0.916 | 0.98(0.80,1.20) | 0.860 | 1.03(0.97,1.08) | 0.306 |
| NO_2_ | Period 1 | 1.04(0.82,1.30) | 0.766 | 0.93(0.87,1.00) | 0.054 | 1.03(0.82,1.29) | 0.792 | 0.94(0.87,1.00) | 0.066 |
|  | Period 2 | 0.92(0.73,1.16) | 0.493 | **0.87(0.81,0.94)** | **<0.001** | 0.95(0.76,1.19) | 0.649 | **0.88(0.82,0.94)** | **<0.001** |
|  | Period 3 | 1.06(0.83,1.36) | 0.622 | **0.92(0.86,0.99)** | **0.024** | 0.97(0.77,1.21) | 0.766 | **0.93(0.86,0.99)** | **0.037** |
|  | Period 5 | 0.90(0.71,1.15) | 0.417 | **0.86(0.80,0.92)** | **<0.001** | 0.97(0.78,1.21) | 0.784 | **0.87(0.81,0.94)** | **<0.001** |
|  | Period 6 | 0.91(0.72,1.16) | 0.453 | **0.86(0.80,0.93)** | **<0.001** | 0.97(0.78,1.20) | 0.760 | **0.87(0.81,0.94)** | **<0.001** |
| SO_2_ | Period 1 | 0.94(0.81,1.08) | 0.392 | **0.94(0.90,0.97)** | **0.002** | 0.98(0.86,1.13) | 0.872 | **0.94(0.90,0.98)** | **0.006** |
|  | Period 2 | 0.98(0.81,1.18) | 0.833 | **0.94(0.90,0.99)** | **0.018** | 0.95(0.80,1.12) | 0.523 | 0.95(0.91,1.00) | 0.058 |
|  | Period 3 | 1.13(0.94,1.36) | 0.196 | 0.99(0.95,1.03) | 0.590 | 1.08(0.91,1.28) | 0.388 | 0.99(0.95,1.03) | 0.647 |
|  | Period 5 | 0.96(0.80,1.17) | 0.717 | **0.94(0.90,0.99)** | **0.014** | 0.97(0.81,1.15) | 0.704 | **0.95(0.90,0.99)** | **0.042** |
|  | Period 6 | 1.00(0.82,1.21) | 0.980 | **0.95(0.90,0.99)** | **0.021** | 0.98(0.83,1.18) | 0.874 | 0.95(0.90,1.01) | 0.053 |
| CO | Period 1 | 0.98(0.82,1.17) | 0.843 | 0.98(0.93,1.03) | 0.494 | 0.90(0.75,1.07) | 0.230 | 0.99(0.94,1.04) | 0.644 |
|  | Period 2 | 1.06(0.87,1.30) | 0.534 | 1.01(0.96,1.06) | 0.774 | 0.97(0.81,1.16) | 0.757 | 1.00(0.95,1.05) | 0.944 |
|  | Period 3 | 0.96(0.80,1.15) | 0.637 | 1.03(0.97,1.09) | 0.335 | 0.93(0.79,1.10) | 0.424 | 0.99(0.95,1.06) | 0.979 |
|  | Period 5 | 1.03(0.85,1.24) | 0.774 | 1.00(0.95,1.05) | 0.961 | 0.96(0.81,1.13) | 0.598 | 1.00(0.95,1.05) | 0.979 |
|  | Period 6 | 1.02(0.85,1.24) | 0.802 | 1.00(0.95,1.05) | 0.900 | 0.95(0.80,1.13) | 0.570 | 0.99(0.95,1.05) | 0.949 |

Note: Results showed in bold indicated statistically significant associations.

PM_2.5_, particulate matter ≤2.5μm, μg/m^3^; PM_10_, particulate matter ≤10μm, μg/m^3^; NO_2_, nitrogen dioxide, μg/m^3^; SO_2_, sulfur dioxide, μg/m^3^; CO, carbon monoxide, mg/m^3^; O_3_, ozone, μg/m^3^.

Model adjusted for female age (<30, 30-34, 35-39, and≥40 years), education level, infertility type (primary or secondary infertility), infertility duration, infertility cause (female factor, male factor, both, and unexplained factor), type of embryo transfer (fresh or frozen embryo transfer), number of embryos transferred (one or two), fertilization method (IVF, ICSI or IVF+ICSI), endometrial thickness, and season of embryo transfer (spring, summer, autumn, or winter).

Period 1, 2, 3, 5, 6 indicate the exposure windows shown in Fig. 1.

Table S2 Association between ambient air pollution exposure and pregnancy outcome and live birth among PCOS and non-PCOS women with the first embryo transfer cycles

|  |  | Clinical pregnancy | | | | Live birth | | | |
| --- | --- | --- | --- | --- | --- | --- | --- | --- | --- |
| Pollutants | Exposure period | PCOS | | Control | | PCOS | | Control | |
|  |  | aOR(95%CI) | P-value | aOR(95%CI) | P-value | aOR(95%CI) | P-value | aOR(95%CI) | P-value |
| PM_2.5_ | Period 1 | 0.95(0.67,1.36) | 0.797 | 1.00(0.88,1.13) | 0.956 | 0.93(0.66,1.31) | 0.687 | 1.02(0.90,1.15) | 0.798 |
|  | Period 2 | 1.14(0.78,1.66) | 0.493 | 0.99(0.86,1.14) | 0.905 | 0.96(0.67,1.39) | 0.849 | 1.01(0.87,1.16) | 0.883 |
|  | Period 3 | 0.96(0.67,1.37) | 0.807 | 1.01(0.88,1.15) | 0.866 | 1.02(0.71,1.45) | 0.933 | 1.01(0.88,1.16) | 0.841 |
|  | Period 5 | 1.17(0.79,1.73) | 0.422 | 1.00(0.87,1.16) | 0.957 | 0.99(0.68,1.44) | 0.953 | 1.02(0.88,1.18) | 0.807 |
|  | Period 6 | 1.16(0.78,1.73) | 0.460 | 0.99(0.86,1.15) | 0.942 | 0.97(0.67,1.43) | 0.908 | 1.01(0.86,1.17) | 0.938 |
| PM_10_ | Period 1 | 1.09(0.79,1.50) | 0.596 | 1.03(0.92,1.15) | 0.630 | 1.11(0.81,1.51) | 0.517 | 1.03(0.92,1.16) | 0.616 |
|  | Period 2 | 0.96(0.69,1.33) | 0.809 | 1.00(0.90,1.12) | 0.956 | 1.16(0.84,1.60) | 0.373 | 1.00(0.89,1.12) | 0.979 |
|  | Period 3 | 0.93(0.68,1.28) | 0.675 | 0.98(0.88,1.11) | 0.814 | 0.98(0.71,1.35) | 0.885 | 1.01(0.90,1.13) | 0.875 |
|  | Period 5 | 0.95(0.67,1.34) | 0.786 | 1.00(0.89,1.12) | 0.981 | 1.14(0.82,1.60) | 0.428 | 1.00(0.89,1.12) | 0.988 |
|  | Period 6 | 0.94(0.66,1.32) | 0.708 | 1.00(0.89,1.13) | 0.924 | 1.13(0.82,1.58) | 0.452 | 1.01(0.90,1.14) | 0.866 |
| O_3_ | Period 1 | 1.01(0.84,1.23) | 0.901 | 1.01(0.94,1.08) | 0.814 | 1.14(0.95,1.38) | 0.165 | 1.02(0.95,1.10) | 0.630 |
|  | Period 2 | 0.81(0.65,1.02) | 0.072 | 0.98(0.91,1.07) | 0.745 | 0.87(0.70,1.08) | 0.225 | 1.05(0.97,1.13) | 0.260 |
|  | Period 3 | 0.97(0.79,1.20) | 0.786 | 0.94(0.87,1.03) | 0.198 | 1.00(0.82,1.23) | 0.987 | 0.96(0.88,1.04) | 0.357 |
|  | Period 5 | 0.86(0.68,1.08) | 0.200 | 0.98(0.91,1.06) | 0.676 | 0.96(0.76,1.20) | 0.701 | 1.05(0.96,1.13) | 0.277 |
|  | Period 6 | 0.87(0.69,1.09) | 0.222 | 0.98(0.90,1.06) | 0.578 | 0.95(0.76,1.18) | 0.663 | 1.04(0.96,1.12) | 0.366 |
| NO_2_ | Period 1 | 1.06(0.84,1.34) | 0.622 | 0.94(0.86,1.03) | 0.168 | 1.08(0.86,1.37) | 0.487 | 0.93(0.85,1.01) | 0.102 |
|  | Period 2 | 0.97(0.78,1.12) | 0.801 | **0.91(0.83,0.99)** | **0.036** | 0.97(0.79,1.21) | 0.825 | **0.91(0.83,0.99)** | **0.041** |
|  | Period 3 | 0.95(0.76,1.19) | 0.657 | 0.94(0.86,1.02) | 0.155 | 0.98(0.79,1.21) | 0.818 | 0.93(0.85,1.02) | 0.129 |
|  | Period 5 | 0.97(0.78,1.21) | 0.800 | **0.90(0.82,0.98)** | **0.018** | 0.98(0.79,1.23) | 0.873 | **0.90(0.82,0.98)** | **0.023** |
|  | Period 6 | 0.96(0.77,1.19) | 0.695 | **0.90(0.83,0.99)** | **0.028** | 0.97(0.79,1.21) | 0.836 | **0.90(0.82,0.99)** | **0.029** |
| SO_2_ | Period 1 | 0.95(0.82,1.10) | 0.469 | **0.92(0.87,0.97)** | **0.001** | 1.01(0.88,1.17) | 0.845 | **0.92(0.87,0.98)** | **0.004** |
|  | Period 2 | 0.94(0.78,1.12) | 0.494 | **0.94(0.88,0.99)** | **0.038** | 0.94(0.79,1.12) | 0.471 | **0.93(0.87,0.99)** | **0.024** |
|  | Period 3 | 1.14(0.96,1.35) | 0.146 | 0.98(0.93,1.03) | 0.437 | 1.10(0.93,1.29) | 0.286 | 0.97(0.92,1.03) | 0.319 |
|  | Period 5 | 0.94(0.78,1.13) | 0.492 | **0.94(0.88,0.99)** | **0.031** | 0.95(0.79,1.14) | 0.573 | **0.93(0.87,0.99)** | **0.025** |
|  | Period 6 | 0.96(0.80,1.16) | 0.700 | **0.94(0.89,0.99)** | **0.044** | 0.97(0.81,1.16) | 0.718 | **0.93(0.88,0.99)** | **0.030** |
| CO | Period 1 | 0.98(0.82,1.16) | 0.814 | 1.02(0.96,1.09) | 0.539 | 0.92(0.77,1.09) | 0.341 | 1.00(0.94,1.07) | 0.953 |
|  | Period 2 | 0.97(0.81,1.16) | 0.722 | 1.00(0.94,1.07) | 0.885 | 0.92(0.77,1.11) | 0.396 | 1.00(0.94,1.07) | 0.925 |
|  | Period 3 | 0.93(0.78,1.11) | 0.421 | 0.99(0.92,1.06) | 0.803 | 0.92(0.78,1.09) | 0.329 | 0.97(0.91,1.05) | 0.523 |
|  | Period 5 | 0.96(0.80,1.15) | 0.639 | 1.00(0.94,1.06) | 0.998 | 0.91(0.76,1.10) | 0.337 | 0.99(0.94,1.06) | 0.941 |
|  | Period 6 | 0.95(0.80,1.14) | 0.592 | 0.99(0.94,1.06) | 0.948 | 0.91(0.76,1.09) | 0.313 | 0.99(0.93,1.06) | 0.910 |

Note: Results showed in bold indicated statistically significant associations.

PM_2.5_, particulate matter ≤2.5μm, μg/m^3^; PM_10_, particulate matter ≤10μm, μg/m^3^; NO_2_, nitrogen dioxide, μg/m^3^; SO_2_, sulfur dioxide, μg/m^3^; CO, carbon monoxide, mg/m^3^; O_3_, ozone, μg/m^3^.

Model adjusted for female age (<30, 30-34, 35-39, and≥40 years), BMI, education level, infertility type (primary or secondary infertility), infertility duration, infertility cause (female factor, male factor, both, and unexplained factor), type of embryo transfer (fresh or frozen embryo transfer), number of embryos transferred (one or two), fertilization method (IVF, ICSI or IVF+ICSI), endometrial thickness, and season of embryo transfer (spring, summer, autumn, or winter).

Period 1, 2, 3, 5, 6 indicate the exposure windows shown in Fig. 1.

Table S3 The Pearson’s correlation coefficients of ambient air pollutant during six exposure periods among PCOS and non-PCOS women

| Period | Pollutant | PCOS group | | | | | | Control group | | | | | |
| --- | --- | --- | --- | --- | --- | --- | --- | --- | --- | --- | --- | --- | --- |
|  |  | PM_2.5_ | PM_10_ | O_3_ | NO_2_ | SO_2_ | CO | PM2.5 | PM10 | O3 | NO2 | SO2 | CO |
| Period 1 | PM_2.5_ | 1.00 |  |  |  |  |  | 1.00 |  |  |  |  |  |
|  | PM_10_ | 0.89 | 1.00 |  |  |  |  | 0.89 | 1.00 |  |  |  |  |
|  | O_3_ | -0.34 | -0.21 | 1.00 |  |  |  | -0.34 | -0.25 | 1.00 |  |  |  |
|  | NO_2_ | 0.68 | 0.56 | -0.38 | 1.00 |  |  | 0.69 | 0.58 | -0.35 | 1.00 |  |  |
|  | SO_2_ | 0.55 | 0.55 | -0.27 | 0.43 | 1.00 |  | 0.57 | 0.58 | -0.27 | 0.43 | 1.00 |  |
|  | CO | 0.65 | 0.60 | -0.42 | 0.46 | 0.51 | 1.00 | 0.66 | 0.63 | -0.42 | 0.47 | 0.56 | 1.00 |
| Period 2 | PM_2.5_ | 1.00 |  |  |  |  |  | 1.00 |  |  |  |  |  |
|  | PM_10_ | 0.91 | 1.00 |  |  |  |  | 0.89 | 1.00 |  |  |  |  |
|  | O_3_ | -0.34 | -0.22 | 1.00 |  |  |  | -0.31 | -0.21 | 1.00 |  |  |  |
|  | NO_2_ | 0.60 | 0.48 | -0.31 | 1.00 |  |  | 0.63 | 0.48 | -0.23 | 1.00 |  |  |
|  | SO_2_ | 0.58 | 0.57 | -0.25 | 0.36 | 1.00 |  | 0.55 | 0.55 | -0.23 | 0.33 | 1.00 |  |
|  | CO | 0.69 | 0.67 | -0.42 | 0.40 | 0.56 | 1.00 | 0.66 | 0.65 | -0.41 | 0.40 | 0.57 | 1.00 |
| Period 3 | PM_2.5_ | 1.00 |  |  |  |  |  | 1.00 |  |  |  |  |  |
|  | PM_10_ | 0.90 | 1.00 |  |  |  |  | 0.89 | 1.00 |  |  |  |  |
|  | O_3_ | -0.36 | -0.25 | 1.00 |  |  |  | -0.34 | -0.25 | 1.00 |  |  |  |
|  | NO_2_ | 0.64 | 0.54 | -0.39 | 1.00 |  |  | 0.66 | 0.56 | -0.35 | 1.00 |  |  |
|  | SO_2_ | 0.60 | 0.58 | -0.26 | 0.42 | 1.00 |  | 0.55 | 0.54 | -0.25 | 0.39 | 1.00 |  |
|  | CO | 0.70 | 0.64 | -0.40 | 0.45 | 0.61 | 1.00 | 0.69 | 0.65 | -0.41 | 0.48 | 0.57 | 1.00 |
| Period 5 | PM_2.5_ | 1.00 |  |  |  |  |  | 1.00 |  |  |  |  |  |
|  | PM_10_ | 0.91 | 1.00 |  |  |  |  | 0.89 | 1.00 |  |  |  |  |
|  | O_3_ | -0.34 | -0.22 | 1.00 |  |  |  | -0.32 | -0.22 | 1.00 |  |  |  |
|  | NO_2_ | 0.61 | 0.47 | -0.29 | 1.00 |  |  | 0.63 | 0.47 | -0.22 | 1.00 |  |  |
|  | SO_2_ | 0.58 | 0.57 | -0.26 | 0.35 | 1.00 |  | 0.55 | 0.55 | -0.23 | 0.32 | 1.00 |  |
|  | CO | 0.68 | 0.66 | -0.42 | 0.39 | 0.56 | 1.00 | 0.65 | 0.65 | -0.42 | 0.38 | 0.57 | 1.00 |
| Period 6 | PM2.5 | 1.00 |  |  |  |  |  | 1.00 |  |  |  |  |  |
|  | PM10 | 0.91 | 1.00 |  |  |  |  | 0.89 | 1.00 |  |  |  |  |
|  | O3 | -0.34 | -0.22 | 1.00 |  |  |  | -0.32 | -0.23 | 1.00 |  |  |  |
|  | NO2 | 0.60 | 0.46 | -0.28 | 1.00 |  |  | 0.62 | 0.46 | -0.21 | 1.00 |  |  |
|  | SO2 | 0.59 | 0.57 | -0.26 | 0.35 | 1.00 |  | 0.56 | 0.55 | -0.23 | 0.32 | 1.00 |  |
|  | CO | 0.68 | 0.67 | -0.42 | 0.38 | 0.57 | 1.00 | 0.65 | 0.66 | -0.42 | 0.37 | 0.58 | 1.00 |

Note: PM_2.5_, particulate matter ≤2.5μm, μg/m^3^; PM_10_, particulate matter ≤10μm, μg/m3; NO_2_, nitrogen dioxide, μg/m3; SO_2_, sulfur dioxide, μg/m3; CO, carbon monoxide, mg/m3; O_3_, ozone, μg/m3.

Period 1, 2, 3, 5, 6 indicate the exposure windows shown in Fig. 1.

Number of patients

Figure S1 Temporal distribution of participants by year

Mean of six air pollutants exposure

Figure S2A Mean of six air pollutants exposure for PCOS patients during the period of the start of gonadotropin medication to serum HCG test from 2013 to 2019

Mean of six air pollutants exposure

Figure S2B Mean of six air pollutants exposure for non-PCOS patients during the period of the

start of gonadotropin medication to serum HCG test from 2013 to 2019
